# Management strategies for patients with subclinical hypothyroidism: a protocol for an umbrella review

**DOI:** 10.1186/s13643-021-01842-y

**Published:** 2021-11-01

**Authors:** Brenda S. Bauer, Amaya Azcoaga-Lorenzo, Utkarsh Agrawal, Colin McCowan

**Affiliations:** School of Medicine, University of St Andrews, North Haugh, St Andrews, KY16 9TF UK

**Keywords:** Subclinical hypothyroidism, Levothyroxine, Systematic review, Review of systematic reviews, Umbrella review

## Abstract

**Background:**

Subclinical hypothyroidism is a thyroid disorder diagnosed from the laboratory blood test results of otherwise asymptomatic patients. It has been associated with poor cardiovascular outcomes, mortality and progression to overt thyroid hormone deficiency. Current guidelines on the management of subclinical hypothyroidism differ because of conflicting evidence on long-term treatment benefits. Even though there are several existing systematic reviews on its clinical outcomes, no definitive conclusion has been reached yet. As such, a new synthesis could help provide more insight and consensus on this topic. To this purpose, this umbrella review will evaluate and synthesise current evidence on the long-term clinical outcomes of the different management strategies for subclinical hypothyroidism.

**Methods:**

This is a protocol for an umbrella review on the management strategies for subclinical hypothyroidism. We will conduct literature searches in multiple electronic databases (from inception onwards), namely MEDLINE, EMBASE, Scopus, Web of Science, Cochrane Database of Systematic Reviews, JBI Evidence Synthesis, Epistemonikos database, PDQ Evidence and the PROSPERO register. There will be no restriction on the date or language of publication. Additional material will be identified through grey literature searches and citation chaining. Review inclusion criteria will be patients with subclinical hypothyroidism, receiving treatment or monitoring, no restrictions on the comparators used and with cardiovascular events, frailty fractures, quality of life and all-cause mortality as primary outcomes of interest. Two reviewers will independently screen all citations, full-text articles and abstract data on a pre-piloted form in duplicate. Methodological quality (or bias) of included studies will be appraised using AMSTAR-2. Any conflicts that arise will be resolved through discussion or involving a third reviewer. A narrative synthesis will be provided with information presented in the main text and tables to summarise and explain the characteristics and findings of the included reviews. Even so, it is not expected that a meta-analysis will be performed due to review variability. Study limitations and methodological quality assessments will also be reported to provide context for the overall summary of evidence.

**Discussion:**

This review will provide a comprehensive summary of the effects of the pharmacological and non-pharmacological management of subclinical hypothyroidism on specific long-term clinical outcomes. It is anticipated that the findings of this umbrella review will aid in the development of consensus-based clinical recommendations for subclinical hypothyroidism, as well as highlight areas for future research. Review findings will be disseminated primarily through peer-reviewed publications.

**Systematic review registration:**

PROSPERO CRD42021235172

**Supplementary Information:**

The online version contains supplementary material available at 10.1186/s13643-021-01842-y.

## Background

Subclinical hypothyroidism (SCH) is a disorder of the thyroid gland in which blood levels of free circulating hormones are normal, but those of thyroid-stimulating hormone (TSH)—which stimulates thyroid hormone production—are elevated. It can only be diagnosed through laboratory tests, and diagnosed patients are typically asymptomatic [1, 2]. As such, the detection of SCH is often incidental [3, 4], and in approximately 2 to 5% of patients, SCH has been found to progress to overt hypothyroidism [2]. SCH has also been linked to an increased risk of cardiovascular disease and mortality [5, 6], frailty fractures [7], cognitive dysfunction, anxiety and depression [8]. Crucially, however, these associations are based on differing conclusions from observational studies and small randomised trials with relatively brief follow-up periods [9].

SCH is treated through the replacement of thyroid hormone using the drug levothyroxine [10]. Even so, the decision to begin replacement therapy has long been controversial because of conflicting findings on whether treatment is beneficial for long-term outcomes [1, 2]. Recently published guidelines on the management of SCH differ in their recommendations, as a result. One evidence-based guideline recommends applying a TSH threshold of 10 mU/L for prescribing levothyroxine because of potential long-term benefits such as cardiovascular outcomes and symptom improvement [11, 12]. On the other hand, Bekkering et al. [13] considered a systematic review of 21 trials that found minimal to no evidence of clinical benefit from replacing thyroid hormones in SCH [14]. In response, a strong recommendation was issued against treatment for most adult patients, except patients with TSH levels greater than 20 mIU/L and pregnant women [13].

It is widely acknowledged that inadequate research has been conducted on the long-term clinical outcomes of managing SCH, especially as inconsistencies remain in the findings of the studies that have been performed to date [1, 9, 15–17]. Since thyroid hormone replacement is a lifelong treatment, it is vital to investigate how levothyroxine affects health in subclinical disease. Equally important are the clinical effects of follow-up with no treatment—for patients who do not meet treatment thresholds, for example—in determining the optimal timing of treatment, as well as the suitability of certain patient groups to receive treatment.

The umbrella review approach is well-suited to the synthesis of a body of contentious evidence, as it allows for a rigorous and systematic assessment of the literature [18]. We will employ this methodology to summarise and compare systematic reviews of various clinical outcomes of the management strategies of subclinical hypothyroidism, which may be either to prescribe treatment or to monitor the patient with no pharmacological intervention. Specifically, the review questions are:

Q1: What is the impact of levothyroxine treatment on long-term clinical outcomes for patients with subclinical hypothyroidism?

Q2: What is the impact of follow-up without treatment on long-term clinical outcomes for patients with subclinical hypothyroidism?

## Methods

### Protocol development

This protocol was registered in the PROSPERO register [19] as CRD42021235172. The methods described below are based mainly on the ‘Umbrella Reviews’ chapter of the JBI Manual for Evidence Synthesis [20], though some elements—protocol length, referencing style, critical appraisal and data collection tools, in particular—have been adapted for our purposes. The Preferred Reporting Items for Systematic Review and Meta-Analysis Protocols (PRISMA-P) guidelines [21, 22] have been followed in reporting this protocol, for which a completed checklist is provided as an additional file [see Additional file 1].

### Inclusion criteria

It is anticipated that all the systematic reviews obtained for this review will have clearly defined inclusion and exclusion criteria, in keeping with systematic review norms and guidelines. Therefore, it will be possible to apply the following criteria while selecting the relevant literature. A summary of the screening criteria is presented in Table 1.

**Table 1:** Review inclusion and exclusion criteria

	Inclusion criteria	Exclusion criteria
**Participants**	Patients diagnosed with subclinical hypothyroidism, no demographic or etiological restrictions	Pregnant women Children and adolescents Diagnosis of overt hypothyroidism
**Intervention**	Management of SCH, i.e. treatment using levothyroxine or follow-up without treatment	No reported treatment status
**Comparator**	No restriction on comparison or control groups	
**Outcomes**	Cardiovascular outcomes Cerebrovascular outcomes Quality of life measures Frailty fractures All-cause mortality Other reported outcomes (secondary)	No reporting of any of the primary outcomes of interest
**Study design**	Quantitative systematic reviews and meta-analyses of empirical research	Any other study types (e.g. narrative reviews, scoping reviews, qualitative syntheses)
**Review characteristics**	Articles in any language Any period of study or date of publication	

#### Participants

The population of interest is restricted to patients diagnosed with subclinical hypothyroidism, regardless of age, setting and the country in which the studies took place. Reviews relating solely to pregnant women, children and adolescents will be excluded because these are special patient groups with additional clinical considerations.

#### Intervention

Inclusion is restricted to systematic reviews of studies involving the management of SCH, whether (i) using levothyroxine for treatment or (ii) follow-up with no treatment. Studies that do not report the treatment status of participants will not be included.

#### Comparator

Any comparison groups will be eligible for inclusion, depending on whether one was used in the synthesis. Therefore, reviews that compare the effects of treatment against no treatment will be included, as well as those that report findings from only one of the two strategies.

#### Outcomes

The primary clinical outcomes of interest are cardiovascular (e.g. heart disease, heart failure, peripheral vascular disease), cerebrovascular (i.e. stroke), quality of life measures (e.g. Underactive Thyroid-Dependent Quality of Life score, Short-Form 36, Thyroid-Related Quality-of-Life Patient-Reported Outcome Measure), frailty fractures and all-cause mortality. Secondary outcomes (e.g. improvements in clinical symptoms, cognitive dysfunction) will also be included if reported in addition to the above.

#### Study design

Only quantitative systematic reviews and meta-analyses of empirical studies will be eligible for inclusion, regardless of whether the studies were randomised clinical trials or observational. Narrative and scoping reviews, as well as purely qualitative reviews, will be excluded during study selection. Any systematic reviews that include theoretical or opinion articles will also be considered ineligible.

Primary studies will not be considered, even when gaps are identified in the evidence within included systematic reviews.

#### Review characteristics

There will be no limitations on the year of publication or study period to allow for temporal comparisons in study findings. Publications in languages other than English will be included in the first instance; if translation is not possible, they will be excluded, but their details reported.

### Information sources and search strategy

Comprehensive searches will be carried out on multiple electronic databases: MEDLINE, EMBASE, Scopus, Web of Science, Cochrane Database of Systematic Reviews, JBI Evidence Synthesis, Epistemonikos database, PDQ Evidence and the PROSPERO register, from inception onwards. There will be no additional filters based on the date or language of publication.

We will use controlled vocabularies and search terms directly related to the review questions such as ‘treatment’, ‘levothyroxine’ and ‘subclinical hypothyroidism’ which will be modified, as needed, to account for database-specific differences. Search filters will be applied to retrieve only systematic reviews. The MEDLINE search strategy, developed with the assistance of an academic librarian, is shown in Additional file 2.

The reference lists of selected reviews will also be checked for eligible syntheses (backward citation chaining) and Google Scholar used for forward citation chaining. A search will also be performed for grey literature, on the WorldCat and Open Grey databases and Internet search engines.

These searches will be updated in the later stages of the review (i.e. during data synthesis) to identify any relevant systematic reviews that will have been published in the interim.

### Study selection

All the references retrieved from the searches will be imported to EndNote X9 [23] to remove duplicate records. The remaining citations will then be imported to Covidence [24] and screened independently by a set of two reviewers in duplicate—first by titles and abstracts—against the inclusion and exclusion criteria described above. In situations where it is impossible to identify inclusion from the title and abstract alone, these articles will progress to full-text review.

Afterwards, articles that pass through the initial screening will be obtained and read in full to determine their eligibility for inclusion. Any disagreements in study selection will be resolved through discussion or the involvement of a third reviewer to reach a consensus. Updated systematic reviews will be included but treated as a single study to prevent duplication during data extraction. All decisions at this stage will be recorded and presented in a PRISMA flow diagram in subsequent reports.

### Data extraction

A pair of reviewers working independently will use a standardised, pre-piloted form to extract data in duplicate. Specifically, data will be collected on first author, year of publication, reported a protocol, objective(s), reported strategies to search literature, number of databases searched and date of last search, any restrictions (e.g. language, geographic or date), inclusion/exclusion criteria, intervention(s) of interest and comparators, patient population, main outcomes of interest, type of study designs included (e.g. randomised controlled trials, observational studies or both), number of included studies, number of studies reporting data for meta-analyses, effect metric(s) reported (e.g. risk ratio), methods to assess study risk of bias, statistical methods to combine studies, summary meta-analytic estimates including heterogeneity measures, additional analyses (e.g. subgroup analysis or sensitivity analysis), metabias assessment (e.g. publication bias across studies), funding source and conflicts of interest. Where presented, data on the Grading of Recommendations, Assessment, Development and Evaluation (GRADE) rating for individual systematic reviews will also be collected. A complete list of fields to be extracted from included reviews is included in Additional File 3.

Disagreements arising from data extracts will be resolved by discussion with a third reviewer to reach a consensus. Where necessary, review authors will be contacted for further information on incomplete or missing data.

### Quality assessment

The critical appraisal of all selected systematic reviews will be conducted in tandem with data extraction, using the AMSTAR (A MeaSurement Tool to Assess systematic Reviews) tool. This checklist was designed to assess the methodological quality of systematic reviews of randomised trials [25] and is currently in its second version, AMSTAR-2 [see Additional File 4]. In recognition of the increasing number of systematic reviews incorporating data from non-randomised and observational studies, the original checklist was updated, published and subsequently validated [26, 27]. Syntheses are judged on 16 domains, including the suitability of the research question and inclusion criteria, the search strategy, the characteristics and critical appraisal of included studies and publication bias. Most domains are rated either ‘Yes’ or ‘No’ though some have the additional option of ‘Partial Yes’.

Discrepancies in the independent assessments made by each pair of reviewers will be resolved by discussion with a third reviewer to reach a consensus. The results of the quality assessments will be applied in the overall synthesis and presentation of findings so that it will also be possible to compare the included reviews by methodological quality. However, the primary studies from included systematic reviews will not be evaluated individually.

### Data synthesis

Review findings will be synthesised narratively, as it is anticipated that there will be several differences in inclusion criteria, methods of synthesis and outcome measures. Overall outcome measures will be presented in tabular form, accompanied by detailed descriptions of review characteristics and quality assessments.

If there are sufficient data from the included systematic reviews, patient characteristics (e.g. age, sex) and methodological differences (e.g. search strategies, definitions of clinical outcomes) will be used to stratify the findings, to allow for further comparisons in the management options for SCH based on these criteria.

There is a considerable burden involved in performing a meta-analysis of existing systematic reviews, given the likelihood of primary studies being counted more than once [28]. This is because of the complexity of taking each review apart and then combining the results of several individual studies, many of which are likely to have different review questions and inclusion criteria. As such, it is anticipated that a meta-synthesis of included meta-analyses will not be performed; key statistical data will only be summarised.

### Confidence in cumulative evidence

The GRADE ratings described within the included systematic reviews will be reported in this umbrella review. However, it is anticipated that not all studies will report these measures, especially older syntheses published prior to the first GRADE guidelines [29]. For such reviews, no new GRADE assessments will be conducted because they involve an assessment of primary studies. As such, this is beyond the scope of this umbrella review.

## Discussion

This is a protocol outlining the processes through which an umbrella review will be performed. It is anticipated that this review of systematic reviews will be useful in summarising and comparing the syntheses of evidence on the management of SCH. As such, its findings may either aid in the development of, or reinforce future evidence-based clinical guidelines. Furthermore, the review will be useful for the identification of any potential biases or gaps that could explain the contradictions in the literature on this topic. Knowledge gaps identified in the literature can also inform future studies and systematic reviews.

The key strength of this overview will be to provide a comprehensive summary of current evidence on the management of SCH through the application of robust and established methods to source, select, appraise and synthesise existing systematic reviews. This information will be of interest to researchers, clinicians and patients with SCH seeking a high-level overview of the evidence; this will be the first umbrella review on this topic, to the authors’ knowledge.

This type of evidence synthesis—the umbrella review—though useful, is also subject to several limitations. First, inclusion in this review is restricted to systematic reviews, but additional empirical studies on the same topic are likely to have since been published. These new findings would, therefore, not be captured in the scope of this secondary synthesis. For this reason, all searches will be updated at least once, towards completion of the review.

Another potential challenge when applying meta-review methodology is overlap in primary research. Study results included in more than one systematic review can cause misleading findings through a multiplier effect because a specified set of findings would be counted more than once. Therefore, a crucial element of data extraction and the subsequent synthesis will be to identify all primary studies and report all instances of overlap.

A third limitation is the differences in inclusion criteria between included studies that impede more quantitative forms of synthesis when conducting an overview. However, given the aim of this review of systematic reviews to collate and summarise all the synthesised literature on the clinical management of SCH, a descriptive and tabular presentation of findings should suffice.

### Protocol amendments

Any amendments to this protocol in the carrying out of this umbrella review will be documented and reported in both the PROSPERO register and any subsequent publications.

### Dissemination plans

The findings of this umbrella review will be disseminated through publication in peer-reviewed journals, via social media networks and relevant conferences.

## Supplementary Information

**Additional file 1.** PRISMA-P checklist.

**Additional file 2.** MEDLINE search strategy.

**Additional file 3.** Data extraction template.

**Additional file 4.** AMSTAR-2 checklist.

## Data Availability

Not applicable.

## Abbreviations

AMSTAR: A MeaSurement Tool to Assess systematic Reviews
GRADE: Grading of Recommendations, Assessment, Development and Evaluation
IU: International units
PICO: Population, Intervention, Comparator and Outcomes
PRISMA: Preferred Reporting Items for Systematic review and Meta-Analyses
PRISMA-P: Preferred Reporting Items for Systematic Review and Meta-Analysis Protocols
SCH: Subclinical hypothyroidism
TFT: Thyroid Function Test
TSH: Thyroid-stimulating hormone
