# Supplementary material for: Management strategies for patients with subclinical hypothyroidism: a protocol for an umbrella review
Source: Syst Rev. 2021 Nov 1;10:290. doi: 10.1186/s13643-021-01842-y (PMC8561963; doi:10.1186/s13643-021-01842-y)
Supplement: Supplementary file 2 — Additional file 2. MEDLINE search strategy. [file 13643_2021_1842_MOESM2_ESM.docx]

**Additional file 2: MEDLINE search strategy**

|  | **Inclusion criteria** | **Search terms** |
| --- | --- | --- |
| **Population** | Patients with subclinical hypothyroidism  No age restrictions  Limit by pregnancy (screening stage) | 1. exp Hypothyroidism/ 2. exp Thyroid diseases/ 3. hypothyroid*.tw 4. (thyroid? adj3 deficien*).tw 5. (thyroid? adj3 insufficien*).tw 6. (thyroid? adj3 failure?).tw 7. (thyroid? adj3 low adj3 product*).tw 8. (thyroid? adj3 under adj3 product*).tw 9. (thyroid? adj3 underactiv*).tw 10. (thyroid? adj3 hypofunction).tw 11. (thyroid? adj3 d?sfunction*).tw 12. or/1-11 13. (mild* OR sub-clinic* OR subclinic*).tw 14. 12 and 13 15. (tsh adj3 (elevat* or increas* or high*)).tw. 16. (SHT OR SCH).tw 17. 14 or 15 or 16 |
| **Intervention** | Treatment with Levothyroxine | 1. exp Hormone replacement therapy/ 2. (thyroid? adj3 (therapy OR treat*)).tw 3. exp Thyroxine/ 4. (thyroxin* or levothyrox* or levo-thyrox* or l-thyrox* or L-T4 or LT4).tw. 5. or/18-21 |
|  | Follow-up with no treatment | 1. (follow?up OR monitor* OR observ* OR surveil*).tw 2. ((no OR lack*) adj2 (treatment* OR therap* OR intervention*)).tw 3. (untreated OR ('not' adj2 treat*)).tw 4. or/23-25 |
| **Comparator** | NA |  |
| **Outcomes** | Cardiovascular outcomes | 1. Exp Cardiovascular diseases/ OR exp Heart diseases/ OR exp Myocardial ischemia/ OR exp Vascular diseases/ OR exp Arteriosclerosis/ 2. Carotid Intima Media Thickness/ OR Intima-Media Thickness, Carotid/ OR Atherosclerosis/ OR Atheroscleroses/ OR Atherogenesis/ 3. ((cardiovasc* OR vasc* OR cardio* OR cardia* OR heart* OR coronary* OR myocard* OR pericard* OR isch$em*) adj2 (disease? OR event? OR arrest? OR fail* OR mortality)).tw. 4. (myocardi* adj (infarct* OR revascular* OR re-vascular* OR isch$emi*)).tw. 5. (heart attack* OR angina).tw. 6. (morbid* adj5 (cardio* OR cardia* OR heart* OR coronary* OR myocard* OR pericard* OR isch$em*)).tw. 7. peripheral arter* disease*.tw. 8. (emboli* OR arrhythmi* OR thrombo* OR atrial fibrillat* OR atrial flutter* OR tachycardi* OR endocardi* OR (sick adj sinus)).tw. 9. (isch$emi* adj2 (vascular OR heart)).tw. 10. (flow-mediated vasodilat* OR flow-mediated dilat* OR endothelial-dependent vasodilat* OR endothelial-dependent dilat* OR endothelial function$ OR carotid intima-media thickness OR intima-media thickness OR carotid-wall thickness OR carotid atherosclerosis OR C-IMT).tw. 11. (lipid$ OR cholesterol OR triglyceride$ OR LDL OR HDL).tw 12. or/27-37 |
|  | QoL | 1. exp Quality of Life/ 2. quality of life.tw. 3. (QoL OR HRQoL).tw 4. or/39-41 |
|  | Cerebrovascular outcomes | 1. exp Stroke/ 2. exp Ischemic Attack, Transient/ 3. (stroke$ OR apoplexy).tw 4. ((cerebrovasc* OR cerebral vascular OR brain) adj2 (disease? OR event? OR arrest? OR fail* OR mortality OR accident* OR death*)).tw 5. ((brain* OR cerebral OR lacunar) adj2 infarct*).tw 6. (isch$emi* adj2 (transient OR attack* OR cerebral or brain)).tw 7. or/43-48 |
|  | Frailty fractures | 1. exp Frailty/ 2. frail*.tw 3. exp Fractures, bone/ OR fracture$.tw 4. (50 or 51) and 52 |
|  | Mortality | 1. (mortality OR death*).tw |
|  | | |
| **Study design** | Systematic reviews | (SIGN Sys Reviews Filter)   1. Meta-Analysis as Topic/ 2. meta analy$.tw. 3. metaanaly$.tw. 4. Meta-Analysis/ 5. (systematic adj (review$1 or overview$1)).tw. 6. exp Review Literature as Topic/ 7. or/55-60 8. cochrane.ab. 9. embase.ab. 10. (psychlit or psyclit).ab. 11. (psychinfo or psycinfo).ab. 12. (cinahl or cinhal).ab. 13. science citation index.ab. 14. bids.ab. 15. cancerlit.ab. 16. or/62-69 17. reference list$.ab. 18. bibliograph$.ab. 19. hand-search$.ab. 20. relevant journals.ab. 21. manual search$.ab. 22. or/71-75 23. selection criteria.ab. 24. data extraction.ab. 25. 77 or 78 26. Review/ 27. 79 and 80 28. Comment/ 29. Letter/ 30. Editorial/ 31. animal/ 32. human/ 33. 85 not (85 and 86) 34. or/82-84,87 35. 61 or 70 or 76 or 81 36. 89 not 88 |
| **PICOS** |  | 1. 17 and (22 or 26) and (38 or 42 or 49 or 53 or 54) |
| **PICOS + Filter** |  | 1. 90 and 91 |
