# Supplementary material for: Management strategies for patients with subclinical hypothyroidism: a protocol for an umbrella review
Source: Syst Rev. 2021 Nov 1;10:290. doi: 10.1186/s13643-021-01842-y (PMC8561963; doi:10.1186/s13643-021-01842-y)
Supplement: Supplementary file 3 — Additional file 3. Data extraction template. [file 13643_2021_1842_MOESM3_ESM.docx]

**Review citation details**

first author (year)

journal

title

**Review purpose**

question(s)

aim/objectives

**Review methods**

protocol registered

reporting guideline used (e.g. PRISMA)

sources/databases searched

date range of searches

date of last search (if repeated)

synthesis methods/analysis

inclusion criteria: PICO – participants, intervention(s), comparator, outcomes of interest, setting/context

definition/description of the outcome(s) – (by review authors)

exclusion criteria (if explicitly stated)

**Reviews results**

number of included studies

study designs included (e.g. RCT, cohort etc)

country of origin of included studies

total number of participants

study details

- author, year of publication, title, study type
- country
- PICO
- number of participants
- number of controls
- time of follow-up

**Quality appraisal**

- quality appraisal tool used
- quality appraisal rating for included studies
- risk of bias assessment

Quality of evidence (e.g. GRADE)

**Review outcomes**

relevant study

meta-analysis (Y/N)

- number of studies included in meta-analysis
- sub-group analysis criteria

effect size

- measure of effect size {eg, ‘Hedge’s g’, incidence rate ratio (IRR), odds ratio (OR), risk ratio (RR)} of association (preferably unadjusted)}
- the outcome and its CI (eg, '1.35 (95% CI 0.97 to 1.73)')
- statistical significance (P < 0.05)

**Significance/direction**

- p value; I^2^; prediction interval; CI of the largest study; equivalent OR; Egger test; excess significance test

**Overall review findings/conclusions**

**Heterogeneity across included studies**

**Review limitations/potential biases**

**Additional notes/comments**
